# Supplementary material for: Sonographic measurement of normal common bile duct diameter and associated factors at the University of Gondar comprehensive specialized hospital and selected private imaging center in Gondar town, North West Ethiopia
Source: PLoS One. 2020 Jan 23;15(1):e0227135. doi: 10.1371/journal.pone.0227135 (PMC6977745; doi:10.1371/journal.pone.0227135)
Supplement: S1 File — (DOCX) [file pone.0227135.s001.docx]

Confidentiality and informed Consent statement

This is a thesis work by Misganaw Gebrie on sonographic assessment of normal common bile duct diameter and associated factor among patients at university of Gondar comprehensive specialized hospital and private imaging center in Gondar town. This study is very important to assess the normal reference range of the common bile duct diameter which is very neccesiciate during the diagnosis and treatment of disease associated with the hepatobilary system .The study also very important to know the normal range of the diameter of the common bile duct at different age group and also its association with the Sociodemographic and anthropometric characteristics. Being a participant of this study have no any problem that will affect the health of the participant and the confidentiality will be maintained. If you are not interested you can discontinue your participation at any time during the study. So your genuine response of the question is very important for the assessment of the normal range of common bile duct diameter and associated factor. I assure that all of your response will be completely confidential and none of your response will be reported separately to anybody. Remember your name is not recorded and no one will be able to find out who give these response. It is your full right to participate or refuse in this study and you may end this interview at any time without any punishment or benefit lose. There is no any risk associated with participation in this study. There is no incentive to be given being participated in the study. If you need clarification you can ask the investigator.

Are you interested to participate in the study A, Yes B, No
