# Supplementary material for: Sonographic measurement of normal common bile duct diameter and associated factors at the University of Gondar comprehensive specialized hospital and selected private imaging center in Gondar town, North West Ethiopia
Source: PLoS One. 2020 Jan 23;15(1):e0227135. doi: 10.1371/journal.pone.0227135 (PMC6977745; doi:10.1371/journal.pone.0227135)
Supplement: S2 File — (DOCX) [file pone.0227135.s002.docx]

Data collection checklist format

To match with inclusion criteria. Detail history and physical examination will be taken. The English version of History taking format of study subjects.

| Ser.no | Questions | Yes | No |
| --- | --- | --- | --- |
| 1 | Have you experienced sever right upper quadrant pain? |  |  |
| 2 | Have you any previous surgery of gallbladder? |  |  |
| 3 | Have you ever diagnosed to have pancreatic disease? |  |  |
| 4 | Have you ever diagnosed to have gall bladder disease? |  |  |
| 5 | Have you ever experienced upper abdominal surgery? |  |  |
| 6 | Have you ever experienced abdominal trauma on the right side? |  |  |
| 7 | Are you pregnant ?(female only) |  |  |
| 8 | Have you ever diagnosed to have liver disease? |  |  |

* If one of the above questions will be answered” yes” and on ultrasonography if there is any finding that will affect the diameter of the common bile duct, the subjects will be excluded from the study.

Data collection checklist for collecting the data to measure the common bile duct diameter and to identify factor associated with common bile duct diameter in Gondar, 2019. In the checklist the diameter and anthropometric measurements will be properly recorded.

| Id | Sex | Age | Weight in KG | Height in Meter | BMI | Residency | | Diameter of common bile duct in mm |
| --- | --- | --- | --- | --- | --- | --- | --- | --- |
|  |  |  |  |  |  | Rural | Urban |  |
| 1 |  |  |  |  |  |  |  |  |
| 2 |  |  |  |  |  |  |  |  |
| 3 |  |  |  |  |  |  |  |  |
| 4 |  |  |  |  |  |  |  |  |
| 5 |  |  |  |  |  |  |  |  |
| 6 |  |  |  |  |  |  |  |  |
| 7 |  |  |  |  |  |  |  |  |
| 8 |  |  |  |  |  |  |  |  |
| 9 |  |  |  |  |  |  |  |  |
| 10 |  |  |  |  |  |  |  |  |
| 11 |  |  |  |  |  |  |  |  |
| 12 |  |  |  |  |  |  |  |  |
