# Supplementary figures and images for: Sonographic measurement of normal common bile duct diameter and associated factors at the University of Gondar comprehensive specialized hospital and selected private imaging center in Gondar town, North West Ethiopia
Source: PLoS One. 2020 Jan 23;15(1):e0227135. doi: 10.1371/journal.pone.0227135 (PMC6977745; doi:10.1371/journal.pone.0227135)

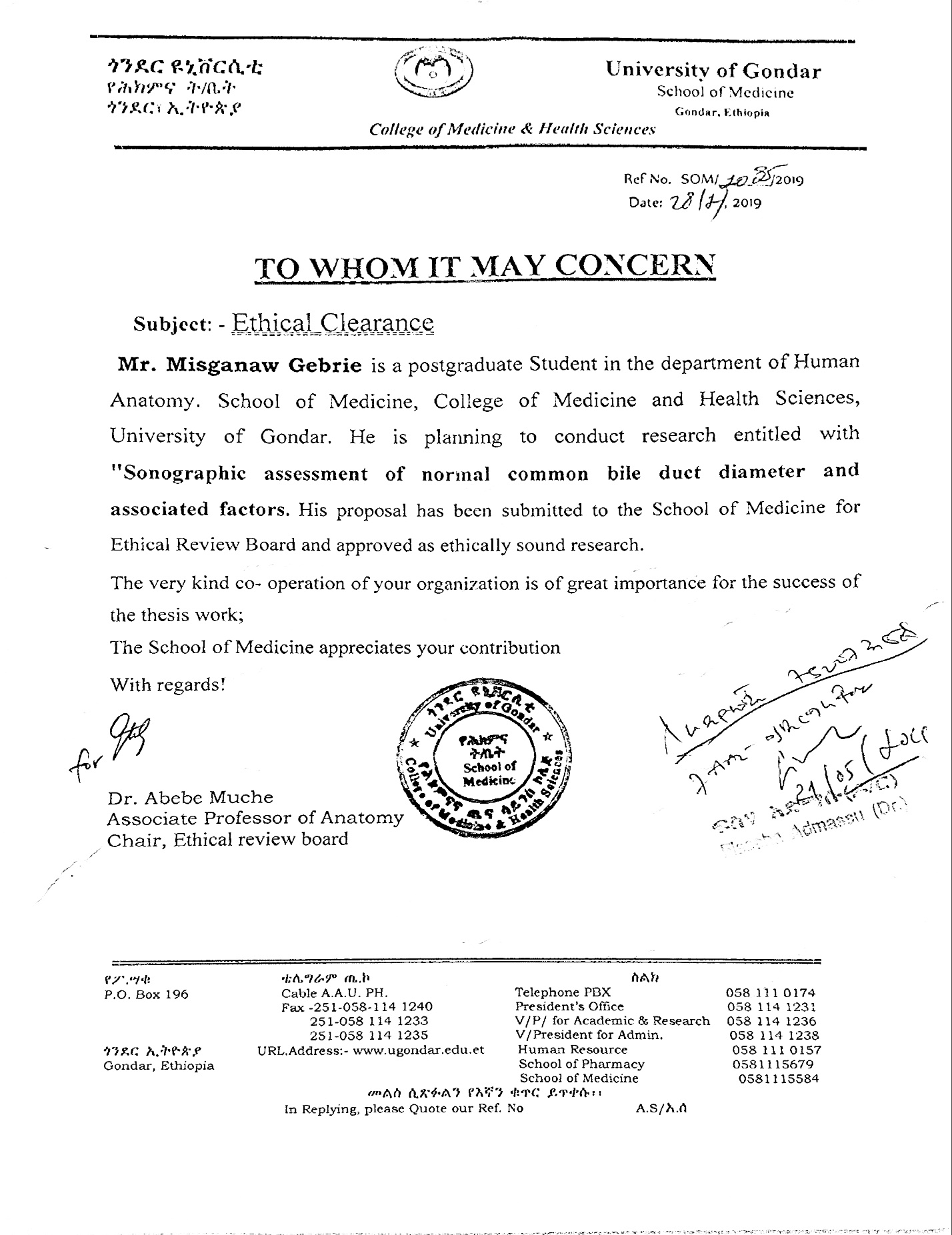

Supplement: S3 File — (DOCX) [file pone.0227135.s003.docx]
