# Supplementary material for: Sonographic measurement of normal common bile duct diameter and associated factors at the University of Gondar comprehensive specialized hospital and selected private imaging center in Gondar town, North West Ethiopia
Source: PLoS One. 2020 Jan 23;15(1):e0227135. doi: 10.1371/journal.pone.0227135 (PMC6977745; doi:10.1371/journal.pone.0227135)
Supplement: Attachment — Submitted filename: Response to editors.docx [file pone.0227135.s004.docx]

Dear PLOS ONE editorial,

First, we would like say thanks for your valuable and interesting comment and suggestion,

Here is the comment provided by your editorial staff and Author’s response

Editorial comment

“Our editorial staff has assessed your submission, and we have significant concerns about the grammar, usage, and overall readability of the manuscript. We believe that the manuscript is much more likely to be accepted if it is easy for the reviewers to read and understand. We, therefore, request that you revise the text to fix the grammatical errors and improve the overall readability of the text before we send it for review. Also, during our internal evaluation of the manuscript, we found significant text overlap between your submission and the following previously published works, some of which you are an author. Please revise the manuscript to rephrase the duplicated text, cite your sources, and provide details as to how the current manuscript advances on previous work.”

Author’s response

The grammatical and spelling errors appeared in this manuscript edited and corrected by a known expert who hasMSc in English and had a good experience. He is working at University of Gondar for the last many years in the same profession. He graduated with BA degree in English language literature from Addis Ababa University and he got his MA degree TEAFL (Teaching English as Foreign Language) in Addis Ababa University. He Provides Teaching English at Addis Ababa University and university of Gondar for over 30 years and Language editor of the journal of health and biomedical sciences at the University of Gondar since its establishment. The manuscript is reviewed, rephrased, and text overlap and redundancy also edited and corrected carefully by the Authors.
