# Supplementary material for: Sonographic measurement of normal common bile duct diameter and associated factors at the University of Gondar comprehensive specialized hospital and selected private imaging center in Gondar town, North West Ethiopia
Source: PLoS One. 2020 Jan 23;15(1):e0227135. doi: 10.1371/journal.pone.0227135 (PMC6977745; doi:10.1371/journal.pone.0227135)
Supplement: Attachment — Submitted filename: Response to reviewer.docx [file pone.0227135.s005.docx]

Dear PLOS ONE editors and reviewers,

We would like to thank for sharing their views and novel scholarly experiences. The comments are very imperative which we strongly believe in improving the manuscript. The point-by-point responses for each of the comments, questions, and the revised manuscript is provided in the attached documents.

Editors and reviewers comments,

1. Please amend either the title on the online submission form (via Edit Submission) or the title in the manuscript so that they are identical.

2. We note you have included a table to which you do not refer in the text of your manuscript. Please ensure that you refer to Tables 6 & 7 in your text; if accepted, production will need this reference to link the reader to the Table.

3. Please ensure that you refer to Figures 1-6 in your text as, if accepted, production will need this reference to link the reader to the figure.

4. Do you have performed chemical analysis (liver functional test) of these subjects to completely rule out any liver alteration?

Author’s response,

1. The comment accepted and the title amended on the online submission and become identical with the title of the main manuscript. The figure files also uploaded to the Preflight Analysis and Conversion Engine (PACE) digital diagnostic tool as TIFF form.

2. Dear PLOSE ONE editorial the comment question raised by your editorial accepted the table and figures which were not previously referred now referred and included in the full text.

3. The file previously included (declaration part) which is out of PLOSE ONE format also excluded in the revised manuscript.

4. Dear reviewers, in our study chemical analysis were not done rather reviewed from the chart and patient having positive results were primarily excluded since it is usually done as a base line investigation. In addition patients having hepatobiliary abnormality that had an effect on our study are excluded by detail history, physical examination and reviewing the medical history including any chemical analysis done and finally through ultrasonographic investigation by a senior radiologist. So Patients having positive results were primarily excluded from our study participants and patients having no chemical analysis like liver function test as a baseline investigation were appropriately examined. During ultrasound investigation patients having positive findings associated with the hepatobiliary system were also excluded. After all this process and exclusion of patients having problem related to the hepatobiliary system, the common bile duct diameter was measured by a single radiologist.
